# Gene Therapy for Neurodegenerative Disease: Clinical Potential and Directions

**DOI:** 10.3389/fnmol.2021.618171

**Published:** 2021-06-14

**Authors:** Xiaolin Zhu, Yu Zhang, Xin Yang, Chunyan Hao, Hubin Duan

**Affiliations:** ^1^Department of Neurosurgery, First Hospital of Shanxi Medical University, Taiyuan, China; ^2^Department of Geriatrics, First Hospital of Shanxi Medical University, Taiyuan, China; ^3^Department of Neurosurgery, Lvliang People’s Hospital, Lvliang, China

**Keywords:** gene editing, neurodegenerative disease, Alzheimer’s disease, Parkinson’s disease, Huntington’s disease, amyotrophic lateral sclerosis

## Abstract

The pathogenesis of neurodegenerative diseases (NDDs) is complex and diverse. Over the decades, our understanding of NDD has been limited to pathological features. However, recent advances in gene sequencing have facilitated elucidation of NDD at a deeper level. Gene editing techniques have uncovered new genetic links to phenotypes, promoted the development of novel treatment strategies and equipped researchers with further means to construct effective cell and animal models. The current review describes the history of evolution of gene editing tools, with the aim of improving overall understanding of this technology, and focuses on the four most common NDD disorders to demonstrate the potential future applications and research directions of gene editing.

## Introduction

Neurodegenerative disease (NDD) refers to a group of chronic disorders characterized by progressive loss of neurons in the brain and spinal cord. Due to technical limitations, our initial understanding of NDD was initially restricted to the pathological manifestations of abnormal protein aggregation, such as Aβ protein in Alzheimer’s disease (AD), huntingtin (HTT) protein in Huntington’s disease, α-synuclein in Parkinson’s disease, and neurofilament in amyotrophic lateral sclerosis. However, treatments targeting abnormal protein levels have constantly faced setbacks in clinical trials. By the end of the 20th century, revolutionary advances in sequencing techniques offered a novel perspective to interpret the mechanisms underlying progression of NDD and gene mutations were identified as drivers of phenotype changes. Thereafter, several studies on NDD at the gene level were conducted. With progression of sequencing methods to third-generation technology, numerous NDD-related mutations and single nucleotide polymorphism (SNP) sites were progressively identified. However, gene mutations cannot explain 100% of NDD cases and sporadic cases exist, even for Huntington’s disease (HD) that is generally considered an autosomal dominant disorder. Thus, in recent years, research focus has expanded from direct gene expression to regulation of expression, which encompasses the fields of transcriptomics, proteomics, and epigenomics.

The concept of gene therapy, first proposed in 1972 (Friedmann and Roblin, 1972), refers to targeted changes in gene sequences through molecular means. In a narrow sense, gene editing is primarily achieved through inducing specific DNA double-strand breaks (DSB) to replace or modify target genes based on the donor sequence. From zinc finger endonuclease to the CRISPR/Cas9 system, the rapidly progressing geneediting field has significant therapeutic potential. The CRISPR/Cas system is particularly outstanding with the advantages of minimal molecular weight and natural existence. Free of artificial design, its convenience and effectiveness has greatly aided in providing insights into the mechanisms underlying NDD, reduced the cost of gene editing, and enabled construction of disease models in both cell and animal systems, in addition to facilitating multiple gene editing valuable for complex diseases such as NDD.

This report provides an overview of the history of gene editing and recent research focus on the four most common NDD disorders, specifically, AD, Parkinson’s disease (PD), HD, and amyotrophic lateral sclerosis (ALS).

## Evolution of Gene Editing

### Homologous Recombination

Homologous recombination is the earliest genome editing technique based on natural DNA damage and the cellular repair system. Chemicals, radiation, and by-products of cellular biological processes, such as reactive oxygen species produced during aerobic respiration, nitrogen compounds produced by inflammatory cells and free radicals generated in hydrolysis reactions, can lead to DNA damage (Jackson and Bartek, 2009). Cells in the human body experience tens of thousands of DNA lesions per day (Lindahl and Barnes, 2000). Among the multiple forms of damage, double-stranded breaks (DSB) are one of the most toxic and difficult to repair (Khanna and Jackson, 2001).

Cells have evolved various mechanisms to repair DSB, two of the most important being homologous recombination (HR) and non-homologous end-joining (NHEJ) (Lieber, 2010). Compared with template-free NHEJ, HR is confined to S and G2 phases (Hustedt and Durocher, 2016; Zhao et al., 2017; Murray and Carr, 2018) while NHEJ is prevalent over the entire cell cycle (Symington and Gautier, 2011). As an important method of allelic exchange, HR also plays a key role in meiosis and mitosis (San Filippo et al., 2008). NHEJ is the dominant repair pathway in mammals and HR competes with other mechanisms (Symington and Gautier, 2011; Kowalczykowski, 2015; Haber, 2016). The repair process of HR is based on multiple pathways, starting from Mre11-Rad50-Nbs1 (MRN) binding to the DSB site followed by 5′-to-3′ resection to generate single-stranded DNA (ssDNA), eventually initiating sub-pathway repair (Mehta and Haber, 2014; Skoneczna et al., 2015; Haber, 2016). HR uses homologous sequences of the sister chromatid as templates in the natural route (San Filippo et al., 2008; Heyer et al., 2010). In genetic engineering, plasmid or viral vector-mediated sequences homologous to DSB ends serve as the new template. However, due to competition with NHEJ, the natural frequency of HR repair alleles in eukaryotes is extremely low (Allen et al., 2002), greatly limiting the efficiency of gene editing, which could be augmented with human intervention in the future.

Evidence indicates that DSBs trigger HR (Rouet et al., 1994; Choulika et al., 1995) and an enzyme designated “meganuclease” or “homing endonuclease” that is naturally present in mitochondria and chloroplasts of microorganisms specifically recognizes 12–30 bp DNA sequences for cleavage without affecting the whole genome (Colleaux et al., 1986; Thierry and Dujon, 1992; Choulika et al., 1994). Owing to the advantages of long recognition sites and 3′ overhang production after DNA cleavage, meganucleases exhibit lower toxicity and better precision than other restriction enzymes (Kc and Steer, 2019). While hundreds of meganucleases have been identified to date, the likelihood of locating the enzyme required for a specific site remains low. On the other hand, since DNA binding and cleavage sites of meganuclease are interspersed in the same domain and are difficult to separate, tailoring the required meganuclease through engineered modifications remains a big challenge (Khan et al., 2018). Overall, the clinical application of meganuclease continues to face technical difficulties (Gaj et al., 2016).

### Zinc Finger Nucleases

In the 1990s, the discovery of Flavobacterium okeanokoites (*Fok*I) enzyme (Sugisaki and Kanazawa, 1981; Li et al., 1992; Kim and Chandrasegaran, 1994) and zinc finger structure (Fegan et al., 1985; Lee et al., 1989) promoted further development of the gene editing technique. Hydrolyzed *Fok*I enzyme (a type IIS restriction endonuclease) in Flavobacterium contains N-terminal DNA-binding and C-terminal domains with non-specific DNA cleavage activity (Li et al., 1992, 1993) that can be easily separated (Waugh and Sauer, 1993). Owing to the modularity of *Fok*I enzyme, engineering is relatively simple.

Zinc finger is an independently folded binding domain that coordinates zinc ions to stabilize the structure. Repeated zinc-binding motifs were first reported in Xenopus transcription factor IIIA (TFIIIA) (Miller et al., 1985). After the first single zinc finger was described in 1989 (Lee et al., 1989), vast complexes were successively identified. Considering Cys2-His2, the most common zinc finger domain as an example, a zinc finger is composed of about 30 amino acids in a conserved ββα configuration (Beerli and Barbas, 2002) and builds contacts with three base pairs in DNA sequences. Zinc finger proteins with conserved sequences are arranged in a certain order followed by attachment of *Fok*I to the 3′ end of the protein, ultimately generating a zinc-finger nuclease (ZFN) consisting of both DNA binding and DNA cleavage domains that recognizes a 9–18 bp sequence (Liu et al., 1997). After binding of ZFN to DNA, the *Fok*I nuclease induces cleavage as a dimer, resulting in DSB. HR and NHEJ are activated to complete gene editing *via* the intracellular DNA repair mechanism (Bitinaite et al., 1998; Smith et al., 2000). Theoretically, ZFNs recognize almost all 64 possible nucleotide triplets but several of these fail in terms of pairing, design and selection (Ramirez et al., 2008; Kim et al., 2010). The specificity and affinity of ZFN is also an issue although optimal fingers have a certain affinity for similar sequences. Increasing the number of zinc fingers can improve specificity and affinity but also raises the issue of inability to access sequences at certain sites, such as those with close chromatin structure and DNA modification (Carroll, 2011). Additionally, ZFNs are reported to exert a significant cytotoxic effect (Khalil, 2020).

### Transcription Activator-Like Effector Nuclease

The discovery of transcription activator-like effector in the plant pathogen Xanthomonas (Boch et al., 2009; Moscou and Bogdanove, 2009) promoted the development of transcription activator-like effector nuclease (TALEN) (Miller et al., 2011), a second-generation nuclease editing technique. The central structure of TALEN protein is a highly conserved sequence of 33–35 amino acids with two variable residues at positions 12 and 13, referred to as repeat variable di-residues (RVD). Each motif relies on RVDs to recognize a single nucleotide (Deng et al., 2012). Similar to ZFN, construction of TALEN is based on the modularity and DNA cleavage function of FokI (Sun and Zhao, 2013). TALEN is easier to design and produce than ZFN but requires about three times as many coding genes. Moreover, its higher molecular weight makes transfection into mammalian cells difficult, especially using virus vectors with limited packaging capacity (Chandrasegaran and Carroll, 2016; Maeder and Gersbach, 2016).

### CRISPR

Clustered regularly interspaced palindromic repeat (CRISPR) was first described in 1987 (Ishino et al., 1987) and its gene editing ability confirmed in human cells in 2013 (Cho et al., 2013). CRISPR exists in 40% bacteria and 90% archaeal genomes and functions as an adaptive immune defense system (Horvath and Barrangou, 2010). The CRISPR system can specifically capture gene sequences adjacent to protospacer adjacent motif (PAM) for cleavage using Cas nuclease into spacer segments derived from the exogenous genome. Spacers are subsequently incorporated into the CRISPR locus of host cells, separated by palindromic sequences, and eventually transcribed to CRISPR RNA (crRNA) with spacer characteristics (Barrangou et al., 2007; Garneau et al., 2010; Horvath and Barrangou, 2010). CrRNA pairs with invading foreign gene sequences in a complementary manner. Simultaneously, Cas nuclease destroys target DNA and completes the entire immune response. By capturing exogenous gene segments from invading phages, viruses and plasmids and incorporating them into host genomic loci, the CRISPR/Cas system sustains acquired immune function (Barrangou et al., 2007; Garneau et al., 2010; Horvath and Barrangou, 2010).

Type II CRISPR/Cas9 composed of Cas9 endonuclease, crRNA and trans-activating crRNA (tracrRNA) is the most commonly used system in genetic engineering (Jinek et al., 2012). Cas nuclease is the core functional element of the CRISPR system. TracrRNA and precursor crRNA (pre-crRNA) bind *via* base pairing, are trimmed by RNaseIII, self-folded into a partial double-stranded RNA structure, and interact with Cas9 to form a complex with DNA cleavage ability. The crRNA-tracrRNA duplex functions as a single guide RNA (sgRNA) that effectively pairs with the target sequence. After binding to the target site, Cas9 undergoes conformational changes and induces DSBs 3–4 nucleotides upstream of PAM (Jinek et al., 2012; Nishimasu et al., 2014). Domains in Cas9 not only interact with the PAM motif but also assist with sgRNA binding to the target sequence (Nishimasu et al., 2014; Barman et al., 2020).

Compared with ZFN and TALEN, the editing horizon of CRISPR is elevated from protein to RNA and technical difficulties from design to assembly are greatly simplified. In terms of target recognition, specificity is higher and binding is more stable. In addition, Cas9 acts as monomer in contrast to *Fok*I, which only cleaves DNA in a dimeric form (Bitinaite et al., 1998; Smith et al., 2000). However, CRISPR does not alter the nature of HR induction through DSBs. In fact, NHEJ is a more prevalent pathway for DSB repair in the entire cell cycle (Chapman et al., 2012). Although NHEJ inhibitors (e.g., Scr7) (Maruyama et al., 2015) and HR promoters (e.g., Cas9-RecA fusion protein) (Cai et al., 2019) are expected to improve the efficiency of HR, CRISPR technology requires further improvement to improve the accuracy of gene editing.

## Latest Developments in Genome Editing

In 2017, a study published in Nature reported a technique denoted “Base Editor” (BE) that achieved single base conversion independently of DSB and HD (Komor et al., 2016; Gaudelli et al., 2018). Based on a complex composed of dCas9 (inactive or dead Cas9) or Cas9n (Cas9 nickase with single-strand DNA incisional enzyme activity), cytosine deaminase (yCD), uracil DNA glycosylase inhibitor (UGI) and sgRNA, BE can achieve four types of accurate base substitution between C/T and G/A (Komor et al., 2016). A new technique known as “Prime Editor” (PE) was reported in 2019 (Anzalone et al., 2019) involving coupling of Cas9 protein with reverse transcriptase. Under guidance of prime editing guide RNA (pegRNA), the PE complex cuts a single strand of DNA at the target site and synthesizes new sequences with the aid of reverse transcriptase. Unpaired sequences in pegRNA are used as templates. The newly synthesized sequence is finally incorporated into the host genome for completion of the gene editing process. PE is free of DNA template and can achieve precise single nucleotide substitutions in sequences inaccessible for BE. While the advent of BE and PE has created new possibilities for gene editing, several concerns remain. For instance, dependence on the Cas9 enzyme limits their recognition window, since Cas9 can only act on sequences adjacent to PAM. BE converts all editable bases in the editing window in a non-specific manner. Moreover, BE can achieve transition of purine–purine and pyrimidine–pyrimidine but not transversion of purine-pyrimidine. In addition, BE and PE only perform edits on single nucleotides and are unable to achieve targeted integration of DNA. The off-target effects of BE and PE in practical applications remain to be established.

In 2019, a new technique using CRISPR-associated transposon (CAST) for DNA transposition was reported in Science (Strecker et al., 2019) and another similar report published in Nature (Klompe et al., 2019). CAST utilizes the ability of Tn7-like transposons to recruit the CRISPR/Cas system in bacteria (Peters et al., 2017). After instrumentalization, Tn7-like transposons can be used for targeted DNA insertion. Independent from DSBs, CAST can effectively carry cargo genes up to 10 kB, which is far superior to the current gene knock-in tool (Hou and Zhang, 2019).

## Alzheimer’s Disease

Alzheimer’s disease is a clinical syndrome characterized by brain amyloid-beta (Aβ) protein deposition in senile plaques (SPs), downstream neuronal degeneration, and tau protein hyperphosphorylation (p-tau) forming neurofibrillary tangles (NFTs) (McKhann et al., 2011). Over the past 20 years, the amyloid cascade hypothesis has dominated research on the pathogenesis of AD. However, identification of mutations within three autosomal dominant genes, specifically, *APP* on chromosome 21 (Goate et al., 1991), *PSEN1* on chromosome 14 (Sherrington et al., 1995), and *PSEN2* on chromosome 1 (Levy-Lahad et al., 1995), has significantly changed research perspective. Subsequent genome-wide association studies (GWAS) have resulted in the identification of another risk gene, ApoE4, and further AD-related SNP sites. These genes encode proteins implicated in various biological processes of AD, which may serve as future editing targets.

Recent GWAS have led to the discovery of dozens of risk loci (Lambert et al., 2013; Kunkle et al., 2019; Vacher et al., 2019; Kunkle et al., 2021). Among these, clear evidence of function has been obtained for ApoE, ABCA7, BIN1, TREM2, SORL1, ADAM10, SPI1, and CR1. In particular, ApoE4 has been extensively characterized in different disease models (Burnham et al., 2020). A recent study showed that Klotho hormone in its biological form reduces risk of AD onset in individuals carrying ApoE4. Moreover, a heterozygous state of KL-VS (KL-VSHET+) genotype was suggested in association with reduced burden of AD and Aβ protein (Belloy et al., 2020). However, no association of KL-VS, the variant of Klotho, with cognitive decline of patients was observed in another clinical study (Porter et al., 2019). The potential contribution of ApoE4 to AD was further examined from multiple perspectives. REST, a central regulator of neural differentiation, is suggested to be related to the ApoE4-induced phenotype (Meyer et al., 2019). Moreover, considering the energy metabolism failure in patients with AD, the ApoE4 genotype may have a regulatory effect on metabolism. These metabolic changes have additionally been linked to gender differences (Arnold et al., 2020). One advantage is that ApoE4 has only one nucleotide difference from its allele ApoE3. Therefore, it is feasible to induce single nucleotide changes, especially with the PE technique. Triggering receptor expressed on myeloid cells 2 (TREM2) is a genetic locus shared by AD and PD. Aggravated neurodegeneration has been detected in TREM2-deleted mice, which may be related to microglial activation (Guo et al., 2019). Another *in vivo* study exhibited transformational value for clinical treatment. Researchers successfully improved Aβ pathology in APP transgenic mice (Nagata et al., 2018; see Table 1).

**TABLE 1:** Pre-clinical studies of gene therapy in neurodegenerative diseases.

Reference	Gene editing tool	Vector	Disease	Target	Animal model	Injections	Results
Nagata et al., 2018	CRISPR/Cas9	px330 plasmid	AD	App 3′-UTR	NL-G-F mice	Microinjected in mice zygotes	Deletion of App 3′-UTR mitigated Aβ pathology in the App KI mice.
Inoue et al., 2018	CRISPR/Cas9	px330 plasmid	PD	p13 exon1	C57BL/6J mice	Injected into the pronuclear stage eggs	Heterozygous p13 knockout prevents motor deficits and loss of dopaminergic neurons in the substantia nigra.
Yang et al., 2017	CRISPR/Cas9	AAV	HD	CAG flanking region	HD 140Q-KI mice	Injected in striatum	Targeted inactivation of CAG repeat could reverse the neuropathological and behavioral phenotypes even in adult mice.
Ekman et al., 2019	CRISPR/SaCas 9	AAV	HD	HTT exon 1	HDR6/2 mice	Injected in striatum	Disruption on HTT reduced mHTT protein, increased lifespan, and protected neurons from death, though lost cells were not restored.
Gaj et al., 2017	CRISPR/Cas9	AAV	ALS	SOD1	G93A-SOD1 mice	Intravenously injected *via* the facial vein	Disruption of mutant SOD1 enhances the survival of spinal cord motor neurons and improves motor function and life span.
Lim et al., 2020	Cytidine base editors	Dual AAV	ALS	SOD1	G93A-SOD1 mice	Injected in the lumbar subarachnoid space	Base editor systems prolonged survival, protected the motor neurons and neuromuscular junctions, slowed the disease progression, decreased muscle denervation.
Duan et al., 2020	CRISPR/Cas9	AAV	ALS	SOD1	G93A-SOD1 mice	Injected in the lateral ventricle (ICV injection)	Deletion of SOD1 delayed motor neuron degeneration and disease onset, and improved the lifespan.

*AD, Alzheimer’s disease; PD, Parkinson’s disease; HD, Huntington’s disease; ALS, amyotrophic lateral sclerosis.*

A number of studies have also focused on the genetic background of AD pathological manifestations. For instance, CHRFAM7A exerts an antagonistic effect on cholinergic receptors in induced pluripotent stem cell (iPSCs) transfected with TALEN (Szigeti et al., 2020). PSENLIN2 is associated with greater amyloid β protein accumulation than PSENLIN1 (Lessard et al., 2019). However, editing the C-terminus of APP *via* CRISPR led to successful reduction of Aβ protein generation in iPSCs (Sun et al., 2019). The Aβ protein-related phenotype was also inhibited by phosphorylation of Threonine 205 (T205) in APP transgenic mice. The post-synaptic mitogen-activated protein kinase (MAPK), p38γ, is further proposed to be involved in regulation (Ittner et al., 2020). Other epigenetically dysregulated loci have been described in a genetic model of *Caenorhabditis elegans*. In another study, mimicking of phosphorylation of Threonine 231 (T231) and acetylation of Lysine 274 (K274) and Lysine 281 (K281) in *C. elegans* was associated with age-related reduction in touch sensation and neuronal morphological abnormalities (Guha et al., 2020).

In addition to the loci that have been extensively investigated, other risk loci identified by GWAS remain to be validated in cell/animal models. A number of studies have included peripheral tissues (such as skin tissue) for analysis. However, the pathogenic significance of these newly identified genes in AD remains to be confirmed (Gerring et al., 2020).

## Parkinson’s Disease

Parkinson’s disease, another important age-related chronic progressive neurodegenerative disorder, is characterized by aggregation of α-synuclein protein. Numerous studies have focused on PD patients with family history-specific mutations in LRRK2, PARK2, DJ-1, PINK1, and SNCA (Sundal et al., 2012). SNCA is directly related to expression of α-synuclein and one of the most significant prediction sites for sporadic PD (Ferreira and Massano, 2017). Mutation and triplication of SNCA A53T affects nucleocytoplasmic transport mediated by α-synuclein (Chen V. et al., 2020). This regulation has been further confirmed in CRISPR-edited iPSCs (Barbuti et al., 2020). Another newly discovered α-syn SNP site, rs12411216, is reported to regulate the function of glucocerebrosidase, which promotes distribution of α-syn protein (Jiang et al., 2020). An improved SCNA-specific CRISPR technique has been applied to generate a PD cell model (Arias-Fuenzalida et al., 2017). A novel CRISPR-based lentiviral vector has additionally been designed to downregulate transcription and expression though targeted methylation of intron 1 of SNCA (Kantor et al., 2018). Another study showed that cell lines depleted of SNCA present resistance to Lewy pathology (Chen X. et al., 2020).

P13, PINK, and PARKIN are additionally highlighted as therapeutic targets on account of their involvement in regulation of mitochondrial function. Several groups have investigated the effect of PARKIN mutation on expression of PD-related proteins in iPSCs lines (Suda et al., 2018). Decreased expression of P13 is reported to exert neuroprotective effects on genetic PD and toxin-induced PD models. In contrast, overexpression of P13 has been shown to promote the emergence of phenotypes in toxin-induced PD mice (Inoue et al., 2018). Some researchers have proposed references for the construction of a LRRK2-related PD stem cell model through cytogenetic analysis (Vetchinova et al., 2018). Another LRRK2 iPSC model constructed with the TALEN technique could additionally serve as a reference (Ohta et al., 2020). In an interesting study, PARKIN, DJ-1, and ATP13A2 genes were deleted using the CRISPR/Cas system in nigral dopaminergic neurons (DN). Through integration of transcriptome and proteome data, oxidative stress was identified as the common dysregulation pathway of all the isogenic cell lines (Ahfeldt et al., 2020). With elucidation of the molecular mechanisms underlying PD, traditional clinical typing may no longer be applicable. More precise delineation of PD subtypes is required, whereby knowledge of molecular etiology could provide further therapeutic perspectives that may be applicable to all NDD disease types.

A novel mutation in DNAJC6 potentially contributes to early impairment of PD in human embryonic stem cells (hESC) (Wulansari et al., 2021). Moreover, PD-related behavioral deficits have been reported in LIN28A knockout mice (Chang et al., 2019). Similar to AD, several recent GWAS for PD have been conducted (Chang et al., 2017; Nabais et al., 2021). However, the issue of whether these disclosed mutations are valuable for clinical prediction requires further study in cell/animal models.

## Huntington’s Disease

Huntington’s disease, a hereditary neurodegenerative disorder characterized by involuntary dance movements and continuous deterioration of behavior and cognition, is commonly associated with disability and early death. HD is distinguished by neuronal loss and astrocytosis in terms of pathology and progressive brain atrophy on imaging. Confirmation of diagnosis is mainly based on family history, clinical symptoms and genetic mutations. Duplication of CAG trinucleotides on exon 1 of Huntington’s gene (HTT) is associated with occurrence of HD (Horvath et al., 2016). Normal CAG repeats on HTT are less than 27 and complete penetration is accomplished when CAG repetition exceeds 39 (McColgan and Tabrizi, 2018). The proteins encoded by the mutated HTT gene (mHTT) cannot participate in physiological cellular mechanisms like their normal protein counterparts and additionally display cytotoxicity. As a disorder caused by single mutation and single abnormal protein, HD is an ideal environment for application of gene therapy.

The construction of HD cell models with gene editing techniques that can be used to validate the efficacy of therapeutic agents has been described in several articles (An et al., 2012, 2014; Xu et al., 2017; Dunbar et al., 2019; Ooi et al., 2019; Malankhanova et al., 2020a). Earlier studies have reported high calcium influx (Vigont et al., 2021) and ultrastructural synapse defects (Malankhanova et al., 2020b) in a HD cell model. Furthermore, the frequency of ultrastructural synapse defects is related to the number of CAG repeats (Morozova et al., 2018).

In another study, deletion of neuronal mHTT was induced *via* CRISPR/Cas9 in HD140Q-KI mice, which led to a significant reduction in reactive astrocytes and improvement of motor dysfunction in the experimental group (Yang et al., 2017). Targeting on the exon 1 of CAG repeat, another *in vivo* study successfully interfered HTT expression as well (Ekman et al., 2019; see Table 1). Based on current studies, although inactivation of CAG expression can effectively alleviate the HD phenotype, the apoptotic cells cannot be restored. Following the success of the non-allele-specific CRISPR system in the PD mouse model, allele-specific CRISPR was shown to be effective in two studies (Shin et al., 2016; Monteys et al., 2017). In addition to directly targeting HTT mutations, CITP2, which interacts with mutant huntingtin (Fjodorova et al., 2019), was edited. ZFN and TALEN were also applied to correct repeated expansion of CAG (Fink et al., 2016; Zeitler et al., 2019).

## Amyotrophic Lateral Sclerosis

Amyotrophic lateral sclerosis is an adult-onset, fatal neurodegenerative disorder. In this disease, apoptosis of the upper motor neurons (e.g., spinal cord, brain stem, and motor cortex) triggers progressive weakness and atrophy of muscles throughout the body, resulting in paralysis and death within 3–5 years after the onset of symptoms. Similar to many other NDDs, ALS is currently incurable. The major pathogenic genes in ALS have been identified as C9orf72, SOD1 (Rosen et al., 1993), FUS, TARDBP, and TBK1 (Müller et al., 2018). However, ALS does not have strong genetic background due to its most cases are sporadic.

Several cell and animal models targeted on SOD1 have been reported (Gaj et al., 2017; Kim et al., 2020). The adeno-associated virus (AAV)-mediated CRISPR system was applied to disrupt SOD1, leading to decreased expression of SOD1 protein in spinal cord and reduction of muscle atrophy in mice. With improvement of motor function, the average survival time of mice increased by 28–30 days (Gaj et al., 2017). Similar favorable results of SOD1 deletion were reported in other animal studies as well (Duan et al., 2020; Lim et al., 2020). Such *in vivo* studies were summarized in Table 1.

The G4C2 hexanucleotide repeat in C9orf72 is a newly described pathogenic factor (DeJesus-Hernandez et al., 2011; Renton et al., 2011). Its pathogenic mechanism can be complex. Researchers found that the deletion of C9orf72 aggravated the axonal defects and thus increased cell apoptosis (Abo-Rady et al., 2020). While recent studies suggest that this pathogenic expansion can be fully corrected using the CRISPR/Cas system (Ababneh et al., 2020), expression of C9orf72 is also reported to affect efficacy of the gene editing process (Moore et al., 2019) and DSB repair (Andrade et al., 2020). The pathogenic effect may achieved through affecting the GluA Q/R site RNA editing (Konen et al., 2020; Moore et al., 2019) and mitochondrial Ca2+ uptake impairment (Dafinca et al., 2020). Many other risk sites have also been reported, such as KIF5A associating to the cytoskeletal defects in ALS (Nicolas et al., 2018). However, further in-depth studies are currently insufficient on these GWAS-identified sites.

## Discussion

In recent decades, integration of the fields of computer science and biology has fueled the development of bioinformatics, with significant improvements in efficacy of analysis and utilization of sequencing data. For instance, genome-wide analysis facilitates prediction of potential risk loci at low cost, which is valuable for complex diseases. To an extent, computer science has revolutionized the paradigm and efficacy of research in traditional experimental biology. These changes have significant implications for gene editing techniques. Despite the fact that gene editing tools are rapidly evolving in terms of improved ease of use and accuracy, actual editing efficacy remains unpredictable, especially *in vivo*. As an auxiliary discipline, computer science is highly valuable in helping to improve the efficacy of editing tools. To this end, researchers have integrated cell-specific information based on gene expression profiles and biological networks to further develop CRISPR sgRNA design tools and predict the efficacy of the CRISPR/Cas system (Liu et al., 2019). Computer science has become an indispensable part of biological research. With increasingly comprehensive research at the molecular level, the accuracy of interpretation of DNA and RNA sequences depends on the advancement of natural language processing techniques. Based on review of the studies on gene editing tools in NDD, we propose the following transformation of future research patterns: clinical studies provide patient information, computers process the profile and make predictions, experimentalists verify the hypotheses, and the data are collectively used to obtain meaningful conclusions.

From the earliest anti-protein treatment strategy to the gene editing technique, one common feature is the translational gap between human and animal models. Often the performance of therapy in humans does not conform to predictions, which could be attributed to the complexity of the human body. In complex organisms, expression of genes is regulated on a multiple and not linear scale. Similarly, expressed products participate in multiple regulatory mechanisms that form a regulatory DNA-RNA-protein network in the human body. Therefore, the actual results of gene editing are inconsistent due to unknown compensation effects (Sun et al., 2019). However, different types of NDD share common pathways, including mitochondrial dysfunction, cytoskeletal integrity, and DNA repair defects (Chia et al., 2018), suggesting that patient stratification *via* molecular typing or genotyping is valuable for treatment. The next step in NDD analysis is to explain the association between clinical syndromes and molecular pathogenesis. Considering that changes in the neuronal phenotype can be directly detected through knockin/knockout, gene editing tools should significantly enrich our knowledge of regulation networks within neurons. In the foreseeable future, gene sequencing will become a routine procedure that directly impacts clinical practice (Chia et al., 2018).

In general, research on gene editing techniques in neurogenerative disease has primarily centered on cell/animal models to explore the underlying biological mechanisms and involves multiple disciplines including molecular biology, cell reprogramming, computer science, statistics, and multi-omics. The major current challenge for NDD is unknown pathogenesis. Researchers have attempted to explore the pathological changes of NDD at the molecular level, whereby gene editing tools play a significant role in clarifying the gene-phenotype relationships. While gene editing tools have been updated at a rapid pace, their clinical transformation may not be easily achievable in the near future. In addition, digitalization has been explored as a critical research direction, from designing of editing tools to construction of disease models. Further studies on NDD incorporating participants from diverse academic backgrounds with large-scale studies are warranted.

## Author Contributions

XZ: manuscript drafting and review and editing. YZ and XY: conceptualization. CH and HD: supervision and review and editing. All authors contributed to the article and approved the submitted version.

## Conflict of Interest

The authors declare that the research was conducted in the absence of any commercial or financial relationships that could be construed as a potential conflict of interest.
